# Multi-Wavelength Interferometric Absolute Distance Measurement and Dynamic Demodulation Error Compensation

**DOI:** 10.3390/s26092677

**Published:** 2026-04-25

**Authors:** Jiawang Fang, Chenlong Ou, Fengwei Liu, Yongqian Wu

**Affiliations:** 1 State Key Laboratory of Optical Field Manipulation Science and Technology, Institute of Optics and Electronics, Chinese Academy of Sciences, Chengdu 610209, China; fangjiawang24@mails.ucas.ac.cn (J.F.); ouchenlong23@mails.ucas.ac.cn (C.O.); 2 Institute of Optics and Electronics, Chinese Academy of Sciences, Chengdu 610209, China; 3 University of Chinese Academy of Sciences, Beijing 100049, China

**Keywords:** multi-wavelength interference, absolute distance measurement, sine phase modulation, the decimal multiple method

## Abstract

This paper presents an absolute distance measurement system based on three-wavelength synchronous phase-shifting interferometry. A synthetic wavelength chain is established using three semiconductor lasers in an all-fiber Fizeau interferometer. By integrating a piezoelectric transducer (PZT)-driven sinusoidal phase modulation with multi-channel synchronous sampling for phase demodulation, and further combining it with a fractional multiplication method, the proposed system achieves high-precision absolute distance measurement over an extended range. Experimental results demonstrate an unambiguous measurement range of 240 μm, a static measurement precision better than 0.6 nm, and a dynamic displacement measurement accuracy superior to 2 nm in comparison with the reference device. The main error sources of the system, including synthetic wavelength uncertainty, phase measurement uncertainty, and air refractive index uncertainty, are systematically modeled and analyzed. In addition, the influence of dynamic factors, such as PZT nonlinearity, is discussed and compensated. The proposed method provides a robust and high-precision solution for absolute ranging and shows strong potential for applications in industrial precision inspection and optical sensing.

## 1. Introduction

High-precision absolute distance measurement with a wide unambiguous range is of great importance in industrial manufacturing, optical fabrication, and advanced lithography. For example, the co-phasing of large-aperture segmented-mirror telescopes requires nanometer-level accuracy over a measurement range of at least several millimeters, because alignment errors directly degrade imaging performance [1,2,3]. Laser interferometry can provide resolution at the nanometer or even picometer level; however, conventional single-wavelength interferometry is inherently affected by phase ambiguity, which limits its unambiguous range to half of the optical wavelength and therefore restricts it to relative rather than absolute measurements.

To overcome this limitation, multi-wavelength interferometry has been extensively investigated since the late 1970s. Bourdet and Orszag introduced the synthetic-wavelength method in 1979, laying the foundation for experimental absolute ranging based on multi-wavelength interferometry [4]. Since then, a series of advances, including heterodyne and superheterodyne detection, phase-locking techniques, and frequency calibration methods, have significantly improved both the measurement range and accuracy [5,6,7,8,9,10]. More recently, the development of femtosecond optical frequency combs and multi-channel synchronous acquisition techniques has further promoted real-time and high-precision absolute distance measurement [11,12,13,14]. In general, research in this area has evolved along three main directions: extending the unambiguous measurement range, improving phase-demodulation accuracy, and enhancing system integration and real-time capability.

Despite these advances, practical engineering applications still face several challenges, including limited system integration, high implementation cost, insufficient real-time performance, and sensitivity to environmental disturbances. To address these issues, this paper proposes a multi-wavelength phase-shifting interferometric absolute ranging system based on an all-fiber architecture and semiconductor lasers. The proposed system establishes a synthetic-wavelength chain using three wavelengths, employs PZT-based sinusoidal phase modulation together with four-bucket integral demodulation to retrieve the single-wavelength phase, and uses a fractional multiplication method to progressively resolve integer ambiguity and achieve high-precision absolute distance measurement. In addition, the principal error and uncertainty sources of the system are systematically analyzed, and the performance of the proposed method is validated through both static and dynamic experiments.

## 2. Principle

### 2.1. System Design

**Figure 1:**
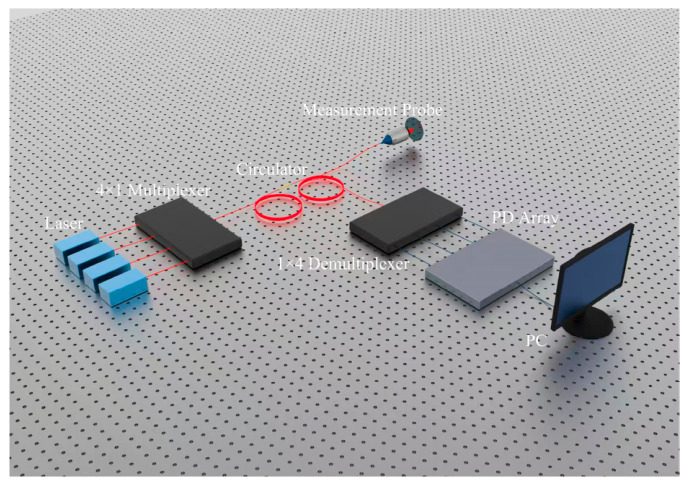
Schematic diagram of multi-wavelength interferometry system.

As shown in Figure 1, the proposed system adopts an all-fiber configuration consisting of three semiconductor lasers, a fiber coupler, a circulator, a PZT-integrated measurement probe, a wavelength demultiplexer, a photodetector array, and a multi-channel synchronous data acquisition card. The three laser beams are combined into a composite beam and routed by the circulator into reference and measurement arms. The measurement beam is focused onto the target surface and reflected back under PZT-driven sinusoidal phase modulation, while the reference beam provides a stable phase reference. The returned interference signals are then demultiplexed into three single-wavelength channels, synchronously detected, and processed in real time to retrieve the phase and absolute distance.

### 2.2. Four-Integration Bucket Method

**Figure 2:**
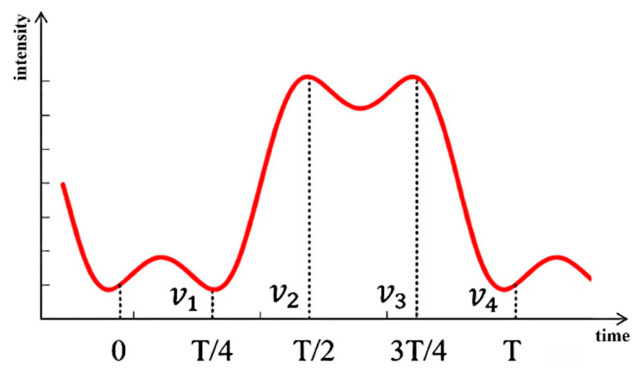
Schematic diagram of the four-integration bucket.

In this research, the four-integration bucket method is employed to extract the unknown phase from the interference signal with high precision. When the test arm is subjected to sinusoidal phase modulation with modulation frequency ω, modulation depth ψ, and initial phase ξ, the interference signal received by the photodetector can be expressed as follows [15]:

(1) $$I\left(t\right)=I_{0}\left(t\right)\left\{1+\gamma \left(t\right)\cos\left[\varphi_{t}\left(t\right)+\psi \sin\left(\omega t+\xi \right)\right]\right\}$$

where $I_{0}$ is the background light intensity; γ is the contrast; and $\varphi_{t}$ is the phase to be measured. To retrieve the unknown phase, the signal is first expanded into a Fourier series using first-kind Bessel functions, as given in Equation (2). Over one modulation period, the signal is integrated over each quarter-cycle according to Equation (3), yielding four integral values, as illustrated in Figure 2. Then, according to Equation (4), these four integral values are linearly combined to construct the orthogonal signals $\Xi_{s}$ and $\Xi_{c}$ [16,17]:

(2) $$\begin{array}{ll} I\left(t\right)= & I_{0}+\gamma J_{0}\left(\psi \right)\cos\varphi_{t}+2\gamma \cos\varphi_{t}\cdot \sum_{n=1}^{\infty }J_{2n}\left(\psi \right)\cos\left[2n\left(\omega t+\xi \right)\right]\\ & -2\gamma \sin\varphi_{t}\cdot \sum_{n=0}^{\infty }J_{2n+1}\left(\psi \right)\sin\left[\left(2n+1\right)\left(\omega t+\xi \right)\right] \end{array}$$

(3) $$\nu_{p}=\int_{\frac{\left(p-1\right)T}{4}}^{\frac{pT}{4}}I\left(t\right)dt,p=1,2,3,4$$

(4) $$\begin{array}{cc} \Xi_{s} & =-\nu_{1}+\nu_{2}+\nu_{3}-\nu_{4}\\ & =\frac{4T}{\pi }A\sin\varphi \cdot \sum_{n=0}^{+\infty }\frac{J_{2n+1}\left(\psi \right)}{2n+1}\cos\left[\left(2n+1\right)\xi \right]=\frac{4T}{\pi }AY_{s}\sin\varphi \\ \Xi_{c} & =-\nu_{1}+\nu_{2}-\nu_{3}+\nu_{4}\\ & =\frac{4T}{\pi }A\cos\varphi \cdot \sum_{n=0}^{+\infty }\frac{J_{4n+2}\left(\psi \right)}{2n+1}\cos\left[2\left(2n+1\right)\xi \right]=\frac{4T}{\pi }AY_{c}\cos\varphi \end{array}$$

where $Y_{s}$ and $Y_{c}$ are the coefficients related to the modulation parameters ψ and ξ. Finally, the phase $\varphi_{t}$ to be measured can be solved by Equation (5). In this system, the modulation depth ψ and the initial phase ξ are determined by pre-calibration.

(5) $$\varphi_{t}=\arctan\frac{Y_{c}\Xi_{s}}{Y_{s}\Xi_{c}}$$

### 2.3. Synthetic Wavelength Chain and Fractional Multiplication

This study employs three laser wavelengths of 1532.68 nm, 1552.52 nm, and 1562.23 nm to construct a three-stage synthetic wavelength chain. The unambiguous range is extended according to Equation (5), thereby enabling absolute distance measurement. Among these stages, the primary single wavelength is 1552.52 nm, the secondary synthetic wavelength is 81.03 μm, and the tertiary synthetic wavelength is 249.78 μm. In general, four-wavelength combinations can expand the unambiguous range by millimeters, and five-wavelength combinations can even expand to the meter level.

(6) $$\Lambda =\frac{\lambda_{1}\lambda_{2}}{\left|\lambda_{1}-\lambda_{2}\right|}$$

The essence of interference ranging is to perform integer counting and fringe subdivision on interference fringes. For each wavelength, there is Equation (7), where L is the distance to be measured, $N_{m}$ and $\varepsilon_{m}$ are the integer order and decimal order of the interference fringes of each wavelength, respectively, and the decimal order can be obtained by phase measurement.

(7) $$L=\left(N_{m}+\varepsilon_{m}\right)\frac{\lambda_{m}}{2}$$

**Figure 3:**
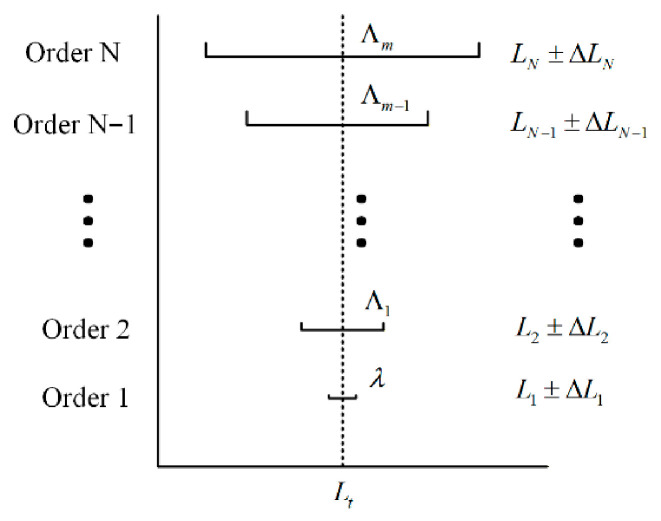
Schematic diagram of the decimal multiplication method.

The fractional multiplication method uses the hierarchical recursion of “long synthetic wavelength-short synthetic wavelength-single wavelength”: Firstly, the unambiguous rough measurement value is obtained at the maximum synthetic wavelength, and then the rough measurement value is used to predict the next integer level candidate set. The unique integer is judged by the residual error of the measured decimal level, and the accuracy is improved step by step until the single wavelength is obtained. Nanoscale results are also obtained. In order to improve robustness, threshold criteria and consistency check are introduced at each level [18]. The step-by-step refinement diagram is shown in Figure 3.

This hierarchical recursion must satisfy the error propagation condition for stepwise refinement, as shown in Equation (8). Therefore, it is necessary to analyze the uncertainty of the measured distance. The expression for the measurement uncertainty is given in Equation (9) (for the synthetic wavelength, the integer order is zero).

(8) $$2\Delta L_{N}<\frac{\Lambda_{N-1}}{2}-2\Delta L_{N-1}$$

(9) $$\Delta L=\sqrt{{\left(\frac{\varepsilon }{2n_{s}}\right)}^{2}\Delta \Lambda_{0}^{2}+{\left(\frac{\Lambda_{0}}{2n_{s}}\right)}^{2}\Delta \varepsilon^{2}+{\left(\frac{\varepsilon \Lambda_{0}}{2n_{s}^{2}}\right)}^{2}\Delta n_{s}^{2}}$$

where $\Delta \Lambda_{0}$ represents the uncertainty of the synthetic wavelength in vacuum; Δε is the fractional uncertainty; and $\Delta n_{s}$ is the refractive index uncertainty of the synthetic wavelength. Equation (9) shows that the error of multi-wavelength absolute distance interferometry mainly derives from three aspects: the uncertainty of the synthetic wavelength, the phase measurement uncertainty, and the uncertainty of the refractive index of air.

**Figure 4:**
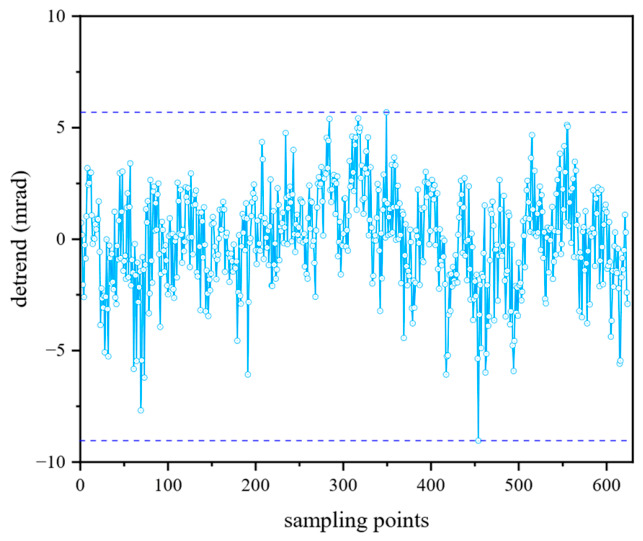
Phase distribution of the primary wavelength.

The uncertainty of the synthetic wavelength is determined by the individual wavelengths that constitute it, as shown in Equation (10). According to the test report provided by the manufacturer, the wavelength stability of the narrow-linewidth laser used in this system can reach 0.4 pm (PV). To measure phase measurement uncertainty, interference signals were collected at a fixed target position. The phase distribution of the primary wavelength is shown in Figure 4 after detrending, and the phase error is calculated to be 2.20 mrad (RMS).

(10) $$\Delta \Lambda_{0}=\sqrt{{\left(\frac{\Lambda_{0}^{2}}{\lambda_{1}^{2}}\Delta \lambda_{1}\right)}^{2}+{\left(\frac{\Lambda_{0}^{2}}{\lambda_{2}^{2}}\Delta \lambda_{2}\right)}^{2}}$$

The synthetic wavelength can be further expressed as follows:

(11) $$\Lambda^{\prime }=\frac{\frac{\lambda_{01}}{n_{1}}\frac{\lambda_{02}}{n_{2}}}{\frac{\lambda_{01}}{n_{1}}-\frac{\lambda_{20}}{n_{2}}}=\frac{\Lambda }{n_{2}-\delta n\frac{\lambda_{02}}{\delta \lambda_{0}}}=\frac{\Lambda }{n_{s}}$$

where Λ′ is the synthetic wavelength in air; *λ*_01_ and *λ*_02_ are two wavelengths in vacuum; *n*_1_ and *n*_2_ are the refractive indices of two wavelengths obtained by the Edlen formula; *δλ*_0_ and *δn* are the difference between the two wavelengths and the corresponding refractive index difference; and *n_s_* is the equivalent refractive index corresponding to the synthetic wavelength. The refractive index of air is constantly affected by atmospheric disturbances, leading to variations in the wavelength and consequently causing measurement errors. It is one of the primary error sources in high-precision distance measurement below the meter level [19,20,21]. To obtain the accurate wavelength of the laser beam during its transmission through air, the refractive index of air must be precisely determined. The Edlén empirical formula can achieve an accuracy of 5 × 10^−8^; therefore, this study employs this formula to calculate the refractive index, as shown in Equation (12) [22,23].

(12) $$n-1=\frac{P\left(n_{tp}-1\right)}{96095.43}\frac{1+P\left(0.601-0.00972T\right)\times 10^{-8}}{1+0.003661T}-e\left(3.8020-\frac{0.0384}{\lambda^{2}}\right)\times 10^{-10}$$

where *T* represents the temperature; *P* represents the atmospheric pressure; and *e* represents the partial pressure of water vapor. Under standard conditions, the total differential of *n* (as shown in Equation (13)) yields the temperature coefficient *K_t_*, the pressure coefficient *K_p_*, and the water vapor partial pressure coefficient *K_e_*, which are −0.951 × 10^−6^ °C^−1^, 0.270 × 10^−8^ Pa^−1^, and −0.379 × 10^−9^ Pa^−1^, respectively. These coefficients indicate that temperature is the main factor causing refractive index drift.

(13) $$dn=\frac{\partial n}{\partial T}dT+\frac{\partial n}{\partial P}dP+\frac{\partial n}{\partial e}de$$

In dry air, the atmosphere has no absorption peak at the 1550 nm band. Therefore, it can be considered that the dispersion characteristics of the atmosphere in this band are uniform, and the refractive index changes linearly in a small wavelength range. Therefore, the refractive index difference can be ignored. It can be considered that the uncertainty of the synthetic wavelength refractive index is approximately the uncertainty of the single-wavelength refractive index.

All experiments in this study were conducted over short distances and within a short duration in a stable laboratory environment. Therefore, the uncertainty of the refractive index of air was not included in the examination of the error propagation conditions. Based on the above analysis, the uncertainty at each stage can be approximately calculated, as shown in Table 1, and is found to satisfy the error propagation conditions.

## 3. Numerical Simulation and Analysis

### 3.1. Optical Probe Design

**Figure 5:**
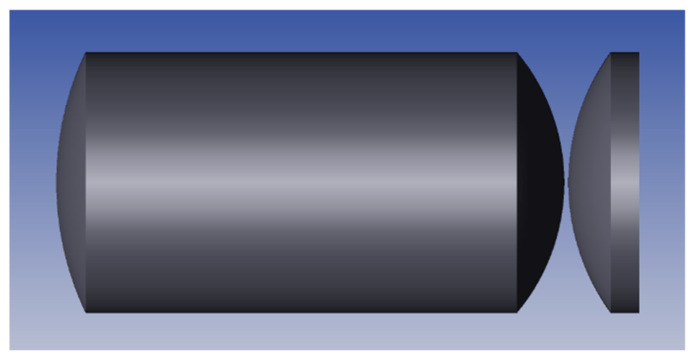
Three-element objective lens used in the probe.

In this study, a three-piece objective lens composed of a double-bonded achromatic lens and a meniscus lens (as shown in Figure 5) was used to effectively correct the aberration and improve the echo coupling efficiency and angle tolerance. The parameters are shown in Table 2.

### 3.2. Multi-Wavelength Interference Algorithm Simulation

**Figure 6:**
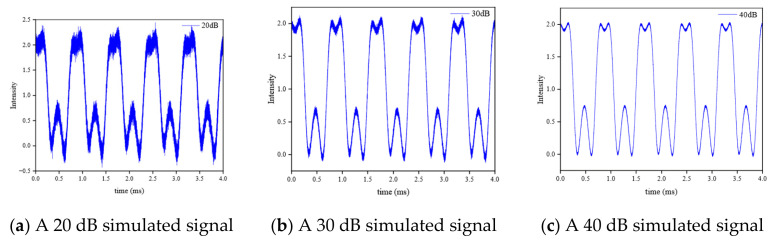

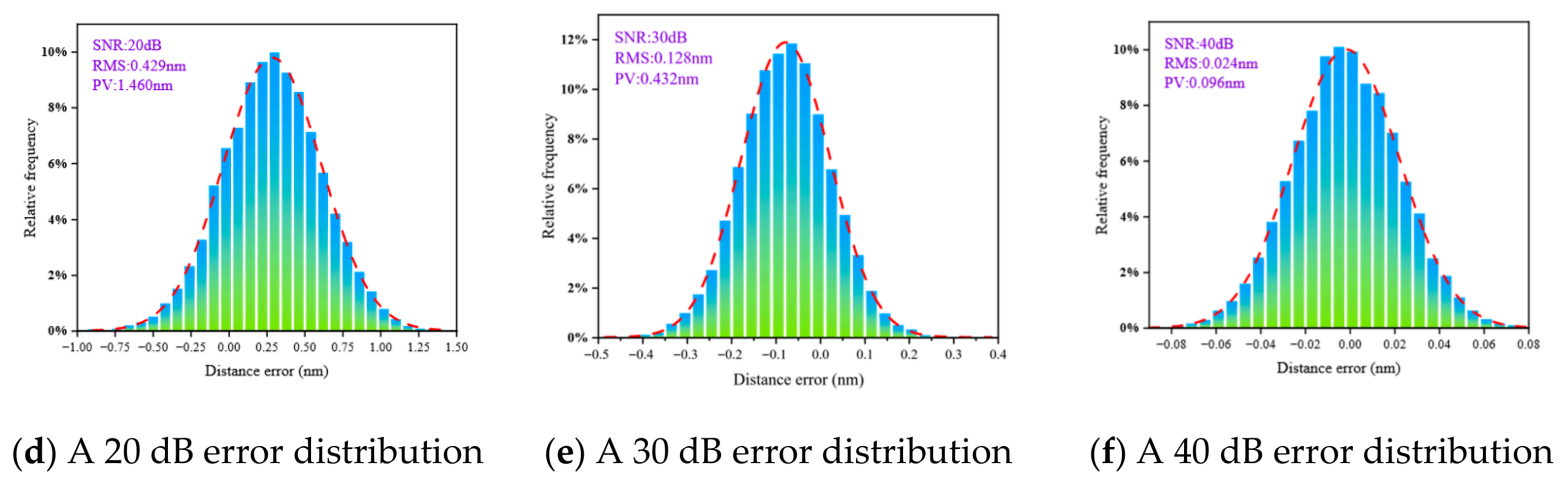
Monte Carlo error distribution.

In order to verify the effectiveness of the synthetic wavelength chain and the fractional multiplication method in the actual noise environment, a Monte Carlo simulation was carried out. The real distance *L* = 16.985 mm is set, and the Gaussian white noise with a root mean square value of 0.4 pm is superimposed on the three vacuum wavelengths. Low-frequency vibration interference and high-frequency random noise are added, and the signal-to-noise ratios are set to 20 dB, 30 dB, and 40 dB, respectively. According to the synthetic wavelength chain and the fractional multiplication method described in this paper, the simulation is repeated 104 times independently. The simulation results are shown in Figure 6. Under the three noise conditions, the distance solution has extremely high accuracy. The results confirm that the proposed algorithm has picometer-level theoretical accuracy and strong robustness.

## 4. Experiment and Results

**Figure 7:**
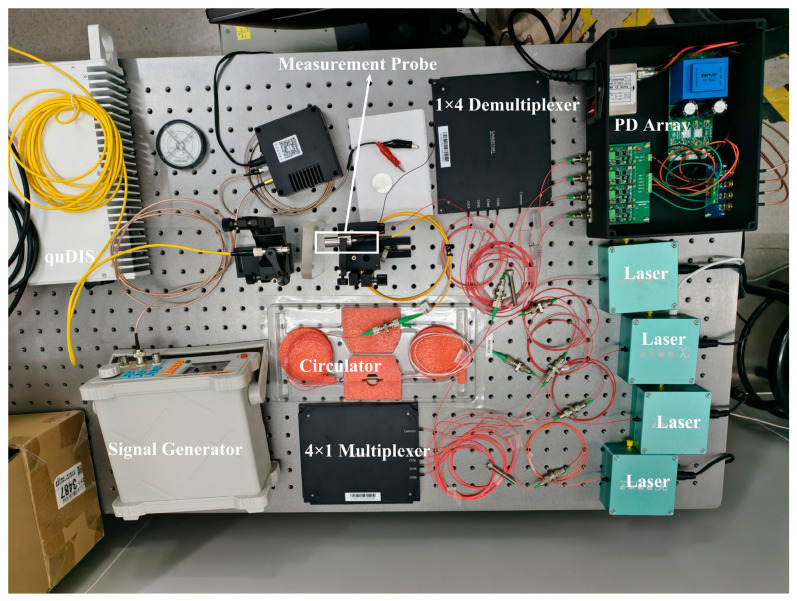
Experimental device diagram.

In order to comprehensively evaluate the performance of the absolute ranging system in various situations, static measurement experiments and dynamic measurement experiments were carried out to test the precision, accuracy and dynamic adaptability of the system. The sampling rate of the experiment is set to 10 MHz, the phase modulation frequency is 1.25 kHz, and the phase modulation depth is 2.45 rad. The actual experimental device is shown in Figure 7.

### 4.1. Static Measurement Experiment

**Figure 8:**
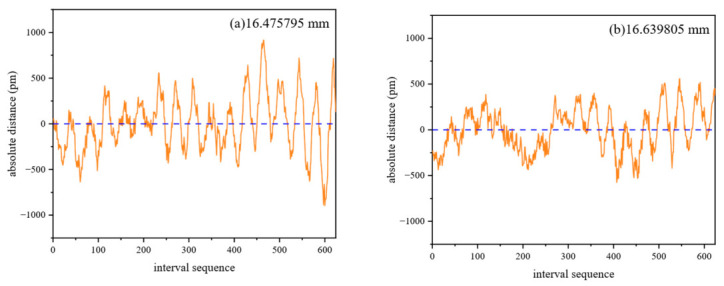
Static measurement results.

The static measurement experiment aims to test the precision of the system in a scene with a fixed target distance. The experiment is carried out on the damping platform to eliminate the interference of external vibration on the measurement results and ensure the reliability of the experimental data. The data of the interference signals of three wavelengths are collected, and the phase measurement is carried out for each wavelength respectively. Then, 3.08 rad, 3.09 rad and 3.01 rad are obtained, respectively, and the phase error is kept within 2%. The results show that the multi-wavelength interference signal of the system has a high degree of consistency in phase, which provides a solid foundation for the subsequent absolute ranging calculation and verifies the synchronization of the three wavelength signals. Finally, the absolute distance is calculated by measuring at different distances. The data are shown in Figure 8 and Table 3 (the data has been decentralized, and the blue line is the actual mean). Table 3 shows that under static conditions, the RMS of each group is less than 0.6 nm, indicating that the system has good precision.

### 4.2. Dynamic Measurement Experiment

**Figure 9:**
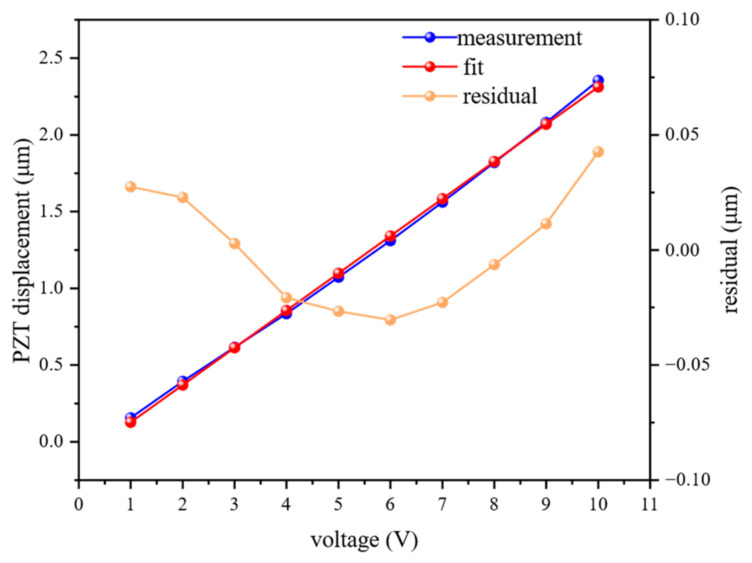
PZT calibration.

The dynamic measurement experiment aims to test the ranging accuracy of the system under target motion conditions. In this study, a signal generator is used to produce a square wave signal to drive the PZT. Under different input voltages, a commercial high-precision picometer-resolution displacement measurement platform, quDIS, is employed to measure the displacement output. The measurement results are shown in Figure 9 and Table 4. Linear fitting is performed on the acquired voltage–displacement data to obtain the voltage–displacement relationship and generate a reference trajectory. Using quDIS as a reference, the ranging accuracy is characterized by comparing the consistency between the measurement data of the proposed system and that of the reference device.

The dynamic measurement experiment aims to test the ranging accuracy and real-time performance of the system in a scene of target motion. The measured mirror is driven by PZT to achieve: (1) linear motion with a stroke of 50 nm; (2) linear motion with a stroke of 900 nm; (3) Sine wave motion with a frequency of 5 Hz and an amplitude of 50 nm; and (4) sine wave motion with a frequency of 5 Hz and an amplitude of 450 nm to simulate dynamic conditions and verify the tracking ability and measurement stability of the system for dynamic targets. Based on the collected dynamic interference signal, the relative displacement between the measured end and the standard mirror is obtained according to the above algorithm.

**Figure 10:**
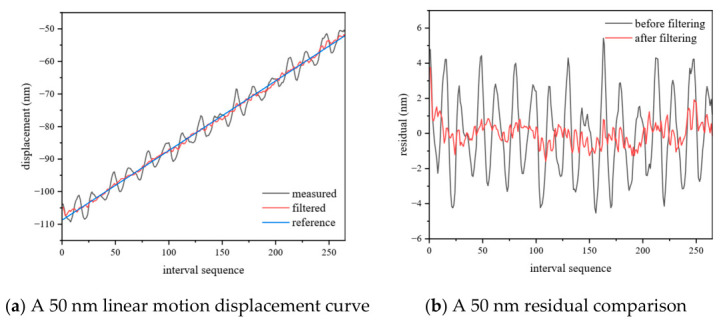

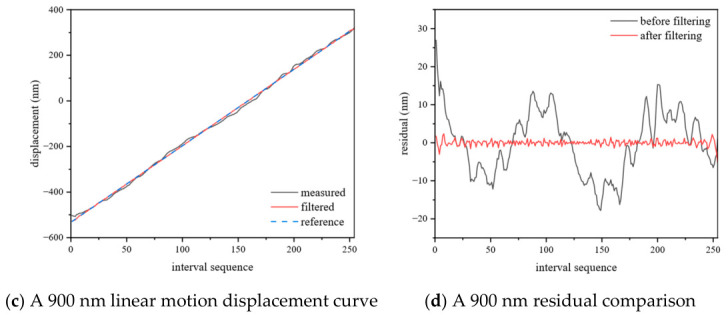
Linear motion.

**Figure 11:**
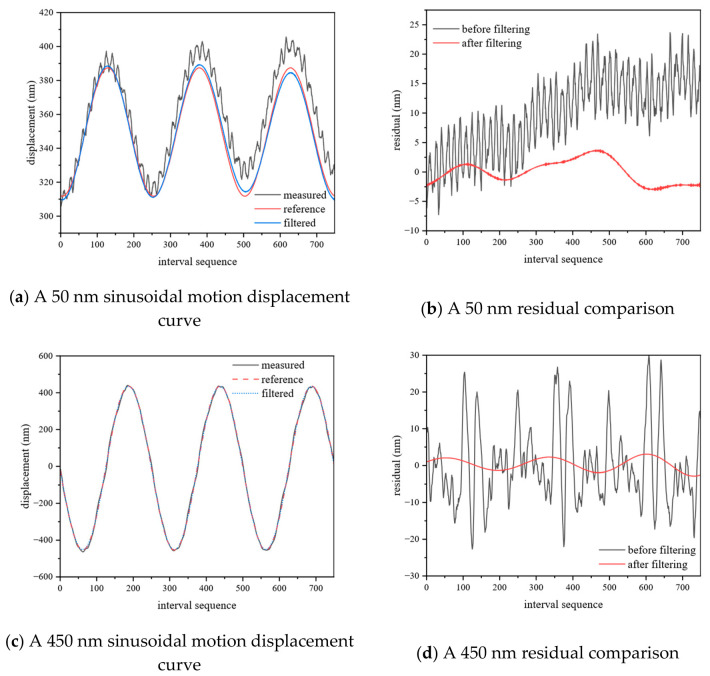
Sine motion.

As shown in Figure 10a,c and Figure 11a,c, the displacements obtained deviate from the ideal motion model and exhibit different noise characteristics: obvious fluctuations in linear motion (1) (2) and sinusoidal motion (3), and obvious distortions at the peaks and valleys of sinusoidal motion (4). The former is due to the fact that environmental vibration is more significant under small amplitude motion and is coupled with the measurement signal. The latter is due to the nonlinear output of PZT itself, and the fact that a non-rigid connection between PZT and the measured mirror aggravates the nonlinear response at the peak acceleration. The noise model of the two can be attributed to the high-frequency component introduced by environmental vibration and the nonlinear response of PZT, which pollutes the signal. Therefore, an error compensation method based on frequency domain filtering is proposed. For the former, the residuals are separated by linear fitting, and then the sharp peaks are suppressed in the Fourier spectrum of the residuals. For the latter, based on the least squares fitting, the approximate dominant frequency is first determined in the frequency domain according to the rough-fine search strategy as the initial value of the fitting, and then the fitting weight is assigned according to the data quality with the goal of minimizing the error, and finally, the optimal dominant frequency is output.

The results after filtering are shown in Figure 10b,d and Figure 11b,d and Table 5. Among them, the PV and RMS of linear motion (2) decreased from 44.774 nm to 7.835 nm and from 8.317 nm to 1.433 nm, respectively, with improvements of 83.2% and 90.6%, respectively, and the improvement of the former being the most obvious. The remaining RMS is improved by more than 70%, which is better than 2 nm, while PV is better than 10 nm. The above results show that the PV and RMS results are significantly reduced after filtering, which proves that the proposed error compensation method can effectively reduce the error (compared with quDIS).

## 5. Conclusions

It should be noted that quDIS does not provide a clear traceability statement or standard uncertainty; therefore, the residuals mentioned above do not represent absolute measurement errors in the strict metrological sense. Furthermore, the reference trajectory was obtained based on static calibration, assuming that the voltage–displacement relationship remains linear and repeatable throughout the entire measurement process. The hysteresis effect of the PZT may introduce residual errors. Despite these limitations, as a commercial high-precision reference device, quDIS still provides a practical and reliable benchmark for evaluating the dynamic measurement performance of the proposed system.

In this paper, a three-wavelength phase-shifting interferometry absolute ranging system based on an all-fiber architecture is developed. The system expands the unambiguous measurement range by synthesizing the wavelength chain, realizing high-precision phase acquisition by using PZT sinusoidal phase modulation and multi-channel synchronous demodulation, and completing the absolute distance step-by-step solution by combining the decimal method. The experimental results show that the unambiguous range of the system is 240 μm, the static measurement precision is better than 0.6 nm, and the dynamic displacement measurement accuracy is better than 2 nm (compared with quDIS). In this paper, the propagation models of main uncertainties such as wavelength stability, phase noise and air refractive index are established. Future work will focus on system miniaturization integration, real-time compensation of environmental disturbances, and application verification in semiconductor manufacturing and aerospace precision metrology.

## Figures and Tables

**Table 1:** Evaluation of error propagation conditions.

Order	1	2	3
uncertainty in wavelength	0.4 pm	1.12 nm	10.4 nm
uncertainty in distance measurement	4.38 nm	234.68 nm	705.07 nm

**Table 2:** Optical probe parameters.

Surface	Radius of Curvature	Thickness	Materials
0	infinite	3.5	
1	8.120	10.530	H-ZF88
2	5.440	3.100	H-ZPK7
3	−5.440	0.100	
4	5.950	1.540	H-ZPK5
5	16.870	16.890	
Image	infinite		

**Table 3:** Static measurement data.

Group	Average/mm	PV/nm	RMS/nm
1	16.475796	1.814	0.305
2	16.639806	1.135	0.233
3	16.934961	1.134	0.226
4	17.176740	2.302	0.567

**Table 4:** PZT calibration data.

Voltage/V	Displacement/μm	Voltage/V	Displacement/μm
1	0.155	6	1.311
2	0.393	7	1.561
3	0.616	8	1.820
4	0.835	9	2.081
5	1.072	10	2.355

**Table 5:** Dynamic measurement data.

Type			PV/nm	RMS/nm
linear	50 nm	before filtering	9.963	2.343
		after filtering	5.346	0.691
	900 nm	before filtering	44.774	8.317
		after filtering	7.835	1.433
sine	50 nm	before filtering	30.936	11.763
		after filtering	6.940	1.942
	450 nm	before filtering	52.579	10.366
		after filtering	5.940	1.678

## Data Availability

The raw data supporting the conclusions of this article will be made available by the authors on request.
